# High-Throughput Peptide Epitope Mapping Using Carbon Nanotube Field-Effect Transistors

**DOI:** 10.1155/2013/849303

**Published:** 2013-07-14

**Authors:** Steingrimur Stefansson, Martha Knight, Hena H. Kwon, Lára A. Stefansson, Saeyoung Nate Ahn

**Affiliations:** ^1^Fuzbien Technology Institute, 9700 Great Seneca Highway, Rockville, MD 20850, USA; ^2^CC Biotech LLC., 9700 Great Seneca Highway, Rockville, MD 20850, USA; ^3^NanoDiagnostics & Devices, Chomdanguiup1-ro, Gumi, Republic of Korea; ^4^Advanced Institutes of Convergence Technology (AICT), Seoul National University, Iuidong 864-1, Suwon, Republic of Korea

## Abstract

Label-free and real-time detection technologies can dramatically reduce the time and cost of pharmaceutical testing and development. However, to reach their full promise, these technologies need to be adaptable to high-throughput automation. To demonstrate the potential of single-walled carbon nanotube field-effect transistors (SWCNT-FETs) for high-throughput peptide-based assays, we have designed circuits arranged in an 8 × 12 (96-well) format that are accessible to standard multichannel pipettors. We performed epitope mapping of two HIV-1 gp160 antibodies using an overlapping gp160 15-mer peptide library coated onto nonfunctionalized SWCNTs. The 15-mer peptides did not require a linker to adhere to the non-functionalized SWCNTs, and binding data was obtained in real time for all 96 circuits. Despite some sequence differences in the HIV strains used to generate these antibodies and the overlapping peptide library, respectively, our results using these antibodies are in good agreement with known data, indicating that peptides immobilized onto SWCNT are accessible and that linear epitope mapping can be performed in minutes using SWCNT-FET.

## 1. Introduction

Antibody epitope mapping involves a precise localization of its binding site on target proteins and is crucial to understanding protective immune mechanisms [1]. Epitope mapping is also of vital importance to both vaccine and drug developers since exact epitope definitions are of paramount importance for patentability. Label-free and real-time epitope mapping using technologies such as surface plasmon resonance (SPR) can dramatically reduce time and effort involved in monitoring antibody binding because it does not require detection components like secondary antibodies and labeled moieties. SPR measures changes in the refractive index of binding events in real time, but the technology requires an expensive and sophisticated optical system [2, 3]. 

Another label-free detection technology uses single-walled carbon nanotubes field-effect transistors (SWCNT-FETs) to detect minute changes in charge of binding events. Single-walled carbon nanotubes (SWCNTs) are manufactured nanomaterials and are essentially two-dimensional graphene sheets forged into elongated tubes while retaining the polyaromatic sp^2^ graphite bonds [4–6]. These nanotubes have a small diameter (~1 nm), consisting solely of a surface where every single carbon atom is in direct contact with the environment. They display unique physical attributes, such as high tensile strength and have excellent semiconductor properties. Exploiting these properties has great potential for producing superior electronic instrumentation and ultra small detectors for biomedical applications [7, 8]. SWCNT-based biodetectors are versatile and have been used to measure antibody, aptamer, or avidin-biotin based capture assays [9–15]. By detecting changes in charge, SWCNT-FET also simplifies and reduces the cost of detection instruments by eliminating optical systems altogether. 

A SWCNT-FET antibody-based detection assay is similar to most immunodetection methods in that the formation of an antibody-ligand complex is measured. Unlike immunodetection methods such as an enzyme-linked immunosorbent assay (ELISA), which requires a labeled secondary binding component to generate a detectable signal, SWCNT-FET detects the change in the electrical properties of an antibody-ligand complex by changes in its impedance (resistance). With proteins, a strong charge transfer to SWCNTs is observed that is believed to be due to the interaction of the protein amino groups with the nanotube surface [16]. 

**Figure 1:**
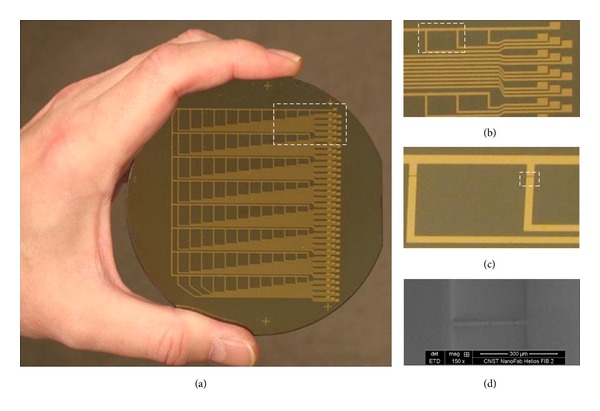
96-circuit SWCNT-FET wafer. The white box in (a) is magnified in (b) and shows details of the contacts for the reader electrode pins. The gold electrodes are 250 *μ*m wide and all 96 circuits are connected to a common source and drain which allows all 96 SWCNT-FETs to be read simultaneously once per second by a signal processor in the reader (not shown). The white box in (b) is magnified in (c), which shows 2 SWCNT channels connected to the source and drain. The white box in (c) is magnified in (d), which shows a scanning EM image of the SWCNT channel. The white bar in (d) indicates 300 *μ*m. Each SWCNT channel is made in between the gold electrodes and has surface area of 5 × 20 *μ*m. (Photography, Dr. Timothy Edberg. SEM, Dr. Joshua Schumacher).

We have previously used SWCNT-FETs printed on 4^″^ semiconducting silica wafers to monitor macromolecular interactions [17–20]. Each of those wafers has 92 independent circuits and was used to measure many biological molecules including IGF-1 [17], glycated human serum albumin [18], *E. coli* O157:H7 [19], and the fibrillar forms of Alzheimer's A*β*_1–40_ and A*β*_1–42_ peptides [20]. However, each circuit on the 92 circuit wafer has to be measured independently, which limits productivity. In an effort to make SWCNT-FET biosensors more applicable to high-throughput assays, we have designed wafers that have circuits in an 8 × 12 (96-circuit) arrangement (Figure 1). The wafers have 8 rows of SWCNT-FET circuits which have the same spacing between them as the 8 rows on 96-well microtiter plates (e.g., 9.0 mm). To fit the 96 channels onto a more economical 4^″^ silica wafer, the 12 columns of SWCNT-FET circuits are compressed by a factor of 2 and have a spacing of 4.5 mm between them instead of the standard 9.0 mm spacing for 96-well microtiter plates. The circuit is a semiconductor element that has three terminals: a source, drain, and gate electrode, which is a configuration similar to that of conventional silicon metal-oxide-semiconductor field-effect transistors (MOS-FETs). The circuits can handle sample volumes between 1 and 10 *μ*L, and the 8 × 12 format makes the circuits accessible to most multichannel pipettors, both manual and robotic. Additionally, the design of the wafers and reader allows real-time reading of all 96 circuits at once. 

To investigate the potential of SWCNT-FET for high-throughput assays, we performed epitope mapping using an overlapping 15-mer peptide library comprising the gp160 HIV-1 viral envelope protein, which is the primary target for generating HIV-1 specific antibodies that can prevent infection [21–23]. 

The 15-mer peptides were coated onto SWCNT-FET circuits and they were scanned using two antibodies with known gp160 epitopes. This peptide library was made using the gp160 sequence of a different HIV strain than that was used to generate the antibodies, so there are some sequence differences. Regardless of these differences, our binding data agrees well with known specificities of these antibodies, and the SWCNT-FET binding data was obtained in minutes.

## 2. Materials and Methods

### 2.1. Materials

The 8 × 12 SWCNT-FET circuits were made on 4^″^ silica semiconductor wafers using standard photolithography and lift-off processes, essentially as previously described [24]. Briefly, an octadecyl-trichlorosilane monolayer pattern on silicon oxide surface of silica wafers was generated by first patterning AZ-5214 photoresist via photolithography, dipping the wafer in the octadecyl-trichlorosilane solution (1 : 500, v/v in hexane) for 3 min, and removing the photoresist patterns using acetone. The SWCNT dispersion was prepared by dispersing purified SWCNTs in 1,2-dichlorobenzene (~0.1 mg/mL) with ultrasonication for 1 hr. The patterned wafer was dipped in the SWCNT solution for 10 sec, rinsed thoroughly with 1,2-dichlorobenzene, and dried with nitrogen gas. This step allowed SWCNTs to be adsorbed selectively onto bare SiO_2_ regions on the wafer, while the octadecyltrichlorosilane blocked nonspecific adsorption of SWCNTs to other regions of the wafer. After assembly of the SWCNTs, electrodes (30 nm Au layer on 10 nm Pd) were fabricated via photolithography and a lift-off process. 

Wafers could be reused by removing existing nanotubes with sonication in 2% SDS followed by extensive washing in deionized water (diH_2_O) and drying. A SWCNT dispersion, enriched in semiconducting type tubes, was provided by Nano-C Inc. (Westwood, MA, USA) in 5 wt% SDS. The new SWCNTs channels were made between the gold electrodes, and SDS was removed by acetic acid. Goat polyclonal antibody against recombinant gp41 (AHP2209) was purchased from AbD Serotec (Raleigh, NC, USA) and was used at 1 : 40 dilution in 25 mM MOPS, pH 7.5. Mouse monoclonal antibody (clone 4E5) to gp120 was purchased from Abnova (Taipei, Taiwan) and was used at 1 : 20 dilution in 25 mM MOPS, pH 7.5. All reagents were used as received. 

### 2.2. Peptide Library

An overlapping 15-mer peptide library comprising the HIV-1 gp160 sequence from sample 037, clone 08 from Uganda (GenBank U09127) was made by Peptide Technologies Corp. (Gaithersburg, MD, USA). This gp160 sequence is not identical to the gp160 used to generate the antibodies. Stock solutions (1 mg/mL) of the peptides were made with 50% acetonitrile/diH_2_O. Optimal coating concentration of the peptides was determined by titrating them onto SWCNT-FET circuits, as described by Stefansson et al. [20]. Coating concentrations were empirically chosen and ranged between 10 and 100 ng/*μ*L (data not shown). Two *μ*L of peptides in diH_2_O were added to each circuit with a multichannel pipettor and were allowed to dry at RT, followed by a brief wash with diH_2_O and drying at RT. Drying the peptides onto the SWCNTs using this method ensured that they were adsorbed onto the SWCNTs.

### 2.3. Measuring Antibody Binding to Immobilized Peptides

To measure antibody binding to peptides coated onto the SWCNT circuits, 4 *μ*L of buffer (25 mM MOPS, pH 7.5) was first added to the circuits for 30 sec to obtain a baseline impedance value, after which 4 *μ*L of antibody dilutions in the same buffer (25 mM MOPS, pH 7.5) was added to the circuits, and impedance was measured for an additional 150 sec. Both 92- and 96-circuit SWCNT-FET wafers were used in this study. Antibody dilutions were made in sample reservoirs and transferred to the wafers with a multichannel pipettor. For accurate determinations, the binding of antibodies to each peptide was measured at least in quadruplicate.

During measurements, a source/drain bias of approximately 100 mV was maintained throughout. The reference electrode is the back (bottom) side of the grounded wafer. Uniformity was not optimized for entire wafers, but circuits used in the assays were evaluated before experiments. The transfer characteristics of the circuit design were previously characterized [17, 24]. The selected SWCNT-FET circuits ranged typically around ten in on-off ratio. The latter is expected to be further increased by improved control of the SWCNT density on the substrate and increased purity of the semiconducting SWCNT relative to the presence of metallic ones. The electrical properties of the antibody-peptide complex were measured using a low-current measurement system developed by MediSourcePlus Inc. and NanoDiagnostics & Devices (South Korea). The 96-circuit wafer reader has pins that make electrical contact to the source and drain electrodes on the SWCNT-FET wafers (Figure 1). The response in the electrical signal is typically in the range of 5 to 40% in normalized resistance, which is the impedance of the antibody normalized to the baseline value obtained by buffer alone.

## 3. Results and Discussion

The mature form of the HIV-1 gp160 envelope protein consists of two noncovalently associated proteins, gp120 and gp41, with the latter containing the membrane spanning region. The gp160 binds to host cell receptors and mediates fusion of viral and target cell membranes. This is a critical step in the infection process and has led to intense efforts to produce specific antibodies against this envelope protein that can inhibit viral entry [26]. However, RNA viruses such as HIV and influenza are highly mutable and can evade therapeutics that target specific amino acid sequences [27, 28]. The sequence variability of the HIV-1 envelope protein is also an issue for epitope mapping [29, 30].

The gp160 peptides were allowed to adsorb to the SWCNT-FET circuits, and the first antibody used in this study was the mouse monoclonal antibody clone 4E5, which was made against a recombinant Fc fusion protein containing a segment of gp120 from HIV-1 CN54, a B/C clade recombinant originally isolated in China [25]. These researchers used overlapping 20-mer peptides for the epitope mapping. The greatest reactivity of the 4E5 monoclonal was found to be against two overlapping 20-mer peptides: ^310^RPGNNTRKSIRIGPGQTFYA^329^ and ^318^SIRIGPGQTFYATGDIIGDI^337^ (GenBank ABY54471.1 numbering). These two 20-mers have a 12 amino acid overlapping sequence containing ^318^SIRIGPGQTFYA^329^, which is likely a vital sequence element recognized by 4E5 [25]. 

**Figure 2:**
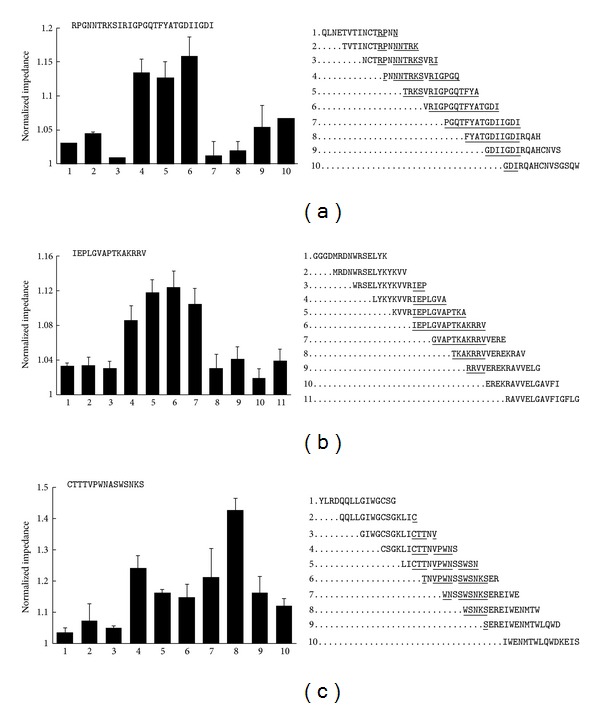
Binding of antibodies to the overlapping gp160 peptide library coated onto SWCNT-FET. Binding of monoclonal antibody 4E5 is shown in (a) and the binding of polyclonal antibody AHP2209 is shown in (b) and (c). The gp160 epitopes that had previously been identified with these antibodies [25, AbD Serotec Datasheet] are shown above the bargraphs. In (a), the two 20-mer sequences identified by Chen et al. [25] are shown as a single contiguous sequence. The sequences of the overlapping 15-mer peptide library used in this study to dissect the previously identified epitopes are shown to the right of the bargraphs. All underlined amino acids show the sequence identity of the previously identified epitopes to the peptide library. Nonunderlined amino acids show where the sequence of the previously identified epitopes and peptide library vary (detailed in the text). The binding of the antibodies to each peptide was performed at least in quadruplicate, and the values represent normalized impedance ± SEM.

There are two amino acid substitutions in the sequence comprising the two 20-mer peptides used by Chen et al. [25] and the 15-mer peptide library used in this study, namely, ^312^G^→^N and ^321^I^→^V. Despite these differences, the greatest reactivity of the 4E5 monoclonal was observed with peptides containing the sequence ^321^VRIGPGQ^327^ (Figure 2(a)) consistently showing the highest impedance, which is in agreement with the results obtained by Chen et al. [25]. Similar to the results reported by Chen et al. [25], we also found that peptides representing sequences either C-terminal or N-terminal to the previously identified epitopes were much less reactive.

The second antibody used in this study was a goat polyclonal antibody (AHP2209), which reacts to recombinant gp41 made from HIV-1 MN (AbD Serotec Datasheet). Results from ELISA-based peptide mapping data of this antibody are provided by the manufacturer and demonstrates high titer reactivity to two distinct and separate regions of the protein, namely, ^491^IEPLGVAPTKAKRRV^505^ and ^604^CTTTVPWNASWSNKS^618^, respectively (UniProt Q70626 numbering, AbD Serotec Datasheet). 

The gp160 sequence used to construct the overlapping 15-mer peptide library used in this study (GenBank U09127) shares roughly 75% amino acid identity with the gp160 that the polyclonal antibody AHP2209 was made against (UniProt Q70626, data not shown). Despite this, the first previously identified epitope, ^491^IEPLGVAPTKAKRRV^505^, is present unaltered in the peptide library, and Figure 2(b) shows that the polyclonal antibody shows the strongest response to peptides containing the sequence ^491^IEPLGVAP^498^. The second region that AHP2209 recognizes is ^604^CTTTVPWNASWSNKS^618^, and it differs from the peptide library in ^607^T^→^N and ^612^S^→^A. But this region in the peptide library is also recognized by the antibody (Figure 2(c)). The results obtained for the second previously identified epitope are more complex than those for the first epitope and suggest that antibody populations in the polyclonal preparation could be binding to two or more sequence elements within the ^609^PWNASWSNKS^618^ sequence (Figure 2(c)). Furthermore, the AbD Serotec Datasheet (http://www.abdserotec.com/product/anti-human-immunodeficiency-virus-1-gp41-antibody-ahp2209.html) states that the AHP2209 displays “moderate activity to multiple other regions on the env polypeptide,” which might contribute to the complex pattern obtained for this antibody. It is also possible that the ^612^S^→^A substitution in the peptide library is affecting the antibody recognition.

We do not believe that the peptide binding to the SWCNTs is affecting the antibody recognition. We found that SWCNTs are quite nonspecific in regard to their ability to bind the 15-mer peptides, especially if the peptides are dried onto the SWCNTs (data not shown). This is supported by phage display studies that have identified a range of peptide sequences that are capable of binding SWCNTs [31–35]. The reason for this lack of a consensus sequence might be due to using nanotube preparations that are of varying purity, diameter, chirality, surface defects, and so forth, for the selection process [33–35]. Nevertheless, the 15-mer peptides immobilized on the SWCNTs are accessible for antibody binding as was observed for a monoclonal antibody binding to A*β* amyloid peptides immobilized on SWCNT-FET [20]. The epitope mapping required relatively high antibody concentrations, which could reflect a lower affinity for partial epitopes represented in the peptide library. Currently, we cannot use SWCNT-FET to ascribe antibody affinities for the peptides but efforts are underway to address this issue.

## 4. Conclusion

SWCNT-FET can facilitate high-throughput epitope mapping in real time, using one antibody and nanogram quantities of peptide. Furthermore, the clonality of the antibody used and the species it is derived from is irrelevant for this SWCNT-FET immunodetection assay.
